# Lacrimal Drainage System and Nasal Cavity Melanoma after Complete Treatment of Conjunctival Melanoma

**DOI:** 10.1155/2024/1034939

**Published:** 2024-02-08

**Authors:** Amirreza Veisi, Zahra Dastborhan, Mohsen Dastmardi, Mozhgan Rezaie Kanavi, Saeid Rezaei

**Affiliations:** ^1^Ophthalmic Research Center, Research Institute for Ophthalmology and Vision Science, Shahid Beheshti University of Medical Sciences, Tehran, Iran; ^2^Shahid Beheshti University of Medical Sciences, Tehran, Iran; ^3^Ocular Tissue Engineering Research Center, Research Institute for Ophthalmology and Vision Science, Shahid Beheshti University of Medical Sciences, Tehran, Iran; ^4^Eye and Skull Base Research Centers, Five Senses Institute, Rasoul Akram Hospital, Iran University of Medical Science, Tehran, Iran

## Abstract

Malignant melanoma of the conjunctiva is a rare tumor of the ocular surface with potential fatal consequences and a high likelihood of recurrence. Although routes for extending the tumor, including local, hematogenous, and lymphatogenous, are pretty straightforward, the indirect extension through free-floating tumoral cells to the nasolacrimal duct is not described thoroughly. We report a case of malignant melanoma of the conjunctiva which presented with local recurrence in the intranasal cavity and lacrimal sac two years after the primary surgery (without involvement of the ocular surface and punctum on the second occasion). However, there was no evidence of distant metastasis on either occasion. This case demonstrates the possible noncontiguous spreading route of melanoma tumoral cells and highlights the need for attention to the surgical technique, and careful follow-up to detect further disease activity.

## 1. Introduction

Conjunctival melanoma, with an aggressive nature and high tendency for spreading and recurrence along with its scarcity, makes it meritorious to be in clinical and research focus.

Although routes for extending the tumor, including local, hematogenous, and lymphatogenous, are pretty straightforward, the indirect extension through free-floating tumoral cells to the nasolacrimal duct is not described thoroughly, and the literature is confined to a few case reports. Due to the importance of management in these patients to prevent mortalities and the lack of content in literature herein, we describe a case of delayed nasolacrimal melanoma without any apparent ocular sign following treated conjunctival melanoma.

## 2. Case Presentation

This case report adhered to the ethical principles outlined in the Declaration of Helsinki.

A 43-year-old female presented to our hospital complaining of a painless dark-colored mass in her right eye. It had developed gradually from a small pigmented spot that appeared six months ago.

**Figure 1:**
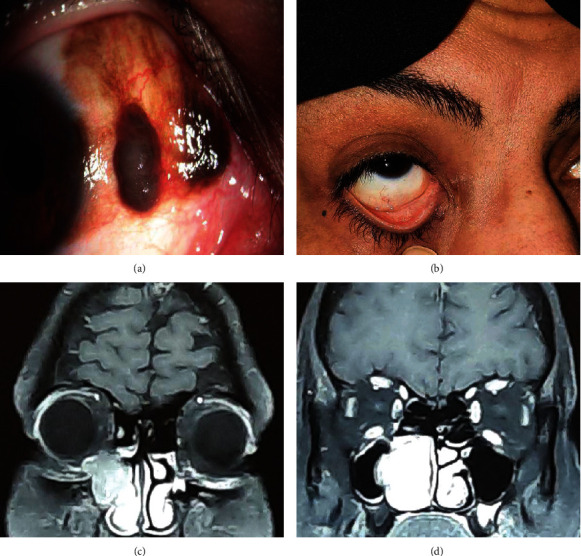
(a) Photoslit of the patient shows two separated lobulated nodular melanotic masses. (b) There is no clinical ocular surface, punctal, medial canthal, and caruncular involvement at the time of intranasal mass diagnosis. (c, d) Orbital and paranasal sinus MRI, coronal section, demonstrating intranasal moderately enhancing mass involving the right-sided nasal cavity and inferomedial part of the right orbit.

Upon examination, two separate globular black-colored masses, each approximately 6 mm in diameter, were found at the 3 o'clock limbus (Figure 1(a)). The lesion was lobulated with spots of superficial hemorrhages and covered by a thin capsule and a few prominent vessels running up to the base of the mass. Caruncular, fornical, and tarsal conjunctiva, as well as superior and inferior punctum, were not involved. The central cornea was clear with peripheral pigmentation, and the best corrected visual acuity was 10/10 OU. The anterior chamber, lens, vitreous, intraocular pressure, and fundus examination were unremarkable. Except for the ulcerative colitis treated with sulfasalazine and ursodeoxycholic acid, there was no other significant medical, surgical, or family history. A systemic evaluation, including liver function tests, abdominopelvic sonography, chest X-ray, brain, and orbital MRI, showed no abnormalities. Besides, B scan ultrasonography showed intact ocular coats, and there was no intraocular extension of the mass.

**Figure 2:**
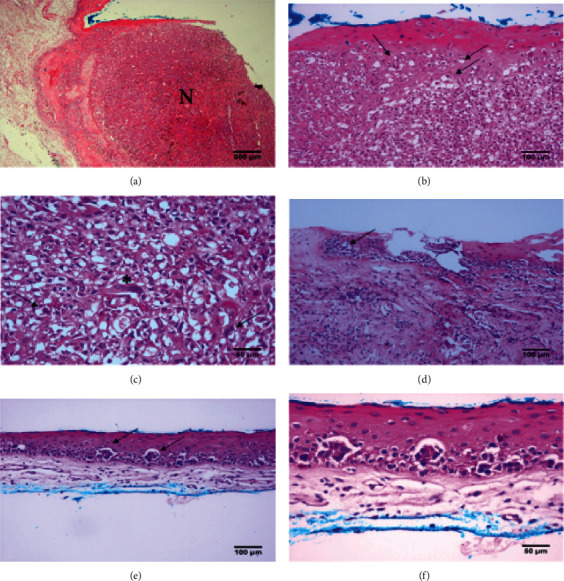
Representative photomicrographs of the presented case with conjunctival melanoma in the context of primary acquired melanosis with atypia (PAM). (a) The nodular proliferation of partially pigmented atypical melanocytes within the conjunctival stroma (N). (b) Nests of intraepithelial atypical melanocytes are highlighted (arrows), and scattered balloon cell transformations are also present. (c) Atypical melanocytes with severe nuclear pleomorphism (asterisk) and a few mitoses (arrows). (d) The presence of nests of atypical melanocytes in the limbal epithelium (arrow). (e (arrows), f) Scattered nests of atypical melanocytes are also present in the surrounding conjunctival epithelium, confirming the diagnosis of PAM.

After the evaluation, the lesion was completely excised with a 3 mm intact margin using the no-touch technique. Subsequently, cryotherapy was applied to the conjunctival margins and scleral bed, and a lamellar keratectomy was performed at the nasal limbus, followed by the reconstruction of the ocular surface using an amniotic membrane. Histopathological examinations revealed nonkeratinized stratified squamous epithelium overlying a nodular proliferation of partially pigmented atypical melanocytes with severe nuclear pleomorphism, distinct nucleoli, a few mitoses, and scattered balloon cell transformations. Nests of atypical melanocytes were also present in the surrounding conjunctival and limbal epithelia (Figure 2). With the diagnosis of conjunctival invasive melanoma in the context of primary acquired melanosis with atypia (PAM), the patient was treated with topical mitomycin-C (MMC) 0.02% (2 weeks on and 2 weeks off) for three months, and at a 12-month regular follow-up, no recurrence was found. However, she missed subsequent follow-up visits.

Two years after the surgery, she faced constant epiphora on the right side and was evaluated by an ENT surgeon this time. She underwent endoscopic dacryocystorhinostomy (DCR) without orbital and paranasal sinus imaging. During endoscopic DCR, the surgeon noticed a black discoloration of the lacrimal mucosa. The specimen taken from the lacrimal duct was reported as a nevus. The symptom-free episode lasted no longer than five months, and epiphora reoccurred, accompanied by epistaxis. Nasal endoscopy revealed a blackish mass in the right-sided nasal cavity. For further investigations, a CT scan and MRI were performed. They showed a mass-like lesion (lobulated moderately enhancing mass) involving the right-sided nasal cavity and inferomedial part of the right orbit (Figures 1(c) and 1(d)). The biopsy result turned out to be melanoma.

After confirming the diagnosis and ruling out distant metastasis with negative results from abdominopelvic and brain MRI and whole-body PET scan, a team performed a complete macroscopic resection of the tumor with globe preservation using an endoscopic approach. The patient underwent adjuvant radiotherapy by an oncologist service and survived for at least 21 months. She died in June 2023 due to metastatic malignant melanoma.

It must be mentioned that the patient's written consent letter was obtained from the study participant.

## 3. Discussion

We presented a case of conjunctival melanoma who experienced lacrimal drainage system and nasal cavity melanoma two years after complete treatment of conjunctival melanoma. The only symptom of LDS melanoma was epiphora, with no concurrent punctual, medial canthal, caruncular, or conjunctival mass or lesion (Figure 1(b)). At the time, the ocular surface and eyelids were free of tumors, at least macroscopically.

Conjunctival melanoma (CM), an uncommon ocular malignancy, accounts for 2% of ocular tumors. Its incidence with an upward trend was reported from 0.45 to 0.8 per million and is associated with significant mortality. It is a highly aggressive cancer with a propensity for spreading and recurring, and given its rarity, it is deserving of greater attention in clinical and research settings [1–3]. Preexisting PAM, de novo generation, and conjunctival nevus could be the origins of CM according to their incidence, respectively[3]. Considering the histopathological investigations, the origin of CM in our case was PAM.

Reports indicate that the overall survival rate for conjunctival melanoma is approximately 75%, with distant spreading being the usual cause of death [4]. Up to one-third of patients develop metastatic disease, and even after complete surgical excision, local recurrence and metastasis are probable [2]. Local recurrence occurs in 26-60% of cases due to residual microscopic disease, and positive margins, palpebral location, primary de novo origin, and excision without supplemental therapy are significant risk factors for local recurrence [5, 6].

Besides, the incidence of distant metastasis is 9-25%; the lung, liver, gastrointestinal tract, and brain are the most common sites of metastasis. Local recurrence history, uncommon location, and more than 2 mm thickness may increase the chance of systemic spread [3].

Additionally, conjunctival melanoma can extend to the orbit, nasolacrimal apparatus, and cornea, and distant metastasis through lymphatic and hematogenous routes is possible [7, 8].

The TNM classification system, which considers primary tumor size, lymph node involvement, and distant metastasis, is associated with survival rates. According to the American Joint Committee on Cancer (AJCC), conjunctival melanomas are divided into malignant tumors of bulbar (T1) and nonbulbar (T2) conjunctiva, tumors involving the orbit, eyelids, sinuses, nasolacrimal apparatus (T3), and brain metastasis (T4) [7, 8]. Our patient presented bulbar conjunctival melanoma with no distant metastasis and intact lymphoid system (T1N0M0) in the first episode. In the second episode, he developed lacrimal drainage system melanoma without any apparent ocular sign and still no distant metastasis (T3N0M0).

Surgical options such as exenteration or removal of the nasolacrimal system are determined based on the extent of the new tumor, and imaging modalities like CT and MRI should be promptly used when nasal and nasolacrimal recurrence is suspected. When the recurrence of melanoma in the nose or lacrimal drainage system is confirmed, a complete evaluation of local and distant metastasis is mandatory.

An incisional biopsy that leads to tumor spreading should be avoided except in particular cases. The “no touch surgery” is a unique popular technique for excising conjunctival tumors. The surgeon should regularly change the instruments and use general anesthesia to avoid contaminating outside of the surgical site with tumor cells [9, 10].

The probability of incomplete excision necessitates supplement treatment with brachytherapy, cryotherapy, and topical chemotherapy like mitomycin-C (MMC 0.02%) to decrease the chance of recurrence by treating intraepithelial tumoral cells [11]. There are conflicting reports on the role of punctal occlusion in the course of MMC therapy, but allowing the medication to get as far as the nasolacrimal duct to reach the tumor extension is a good alternative choice [12].

Following conjunctival melanoma, there are several speculated ways in which melanoma of the ipsilateral nasolacrimal duct may occur, including direct extension, metastasis through the hematogenous and lymphatic system, primary nasolacrimal tumor, and implantation of free-floating tumoral cells in the tear film [13]. The noncontiguous spreading of conjunctival melanoma in the nasolacrimal system was described in a case and named “melanorrhea” [13]. Similarly, our patient developed lacrimal drainage system melanoma following indirect extension of CM tumoral cells. The emergence of the COVID-19 pandemic has negatively impacted access to healthcare providers due to safety issues, lockdowns, and fear of the disease. According to a multicenter study in the UK in which four ocular oncology centers participated, COVID-19 negatively impacts the referral number and diagnosis of certain ocular cancers [14]. Missing cancer screenings and follow-ups could result in delayed diagnoses and consequently lead to more advanced stages and higher mortality rates. In our presented case, missing follow-up sessions started in the era of COVID-19, and we speculated that this factor should be taken into account in managing such cases (Table 1).

A thorough physical examination as well as proper imaging techniques like MRI should be performed when a patient is suspected of conjunctival melanoma and, after that, in each follow-up visit to enhance diagnostic accuracy and help surgical planning. Patient referral to an oncologist should be taken into consideration when the diagnosis is confirmed. A metastatic workup for detecting potential metastatic disease 2 or 3 times a year is advisable [15–17].

Some studies have suggested impression cytology as an alternative and feasible diagnostic tool to evaluate the ocular surface and the inner portion of the punctum during follow-up visits [18]. The use of a punctum plug during surgical resection should be considered to minimize the risk of local recurrence in cases of conjunctival melanoma. Another study by Peck et al. reported the spread of conjunctival melanoma to LAD, several years after successful treatment. The mechanism is still unclear, but ophthalmologist should consider the potential of conjunctival melanoma to involve LAD. This condition specially occurs in the tumor of medial conjunctiva, plica semilunaris, and caruncle. The risk of spreading should be considered before initial treatment [13].

In our patient, the tumor reoccurrences after missing follow-up sessions and is probably introduced into the nasal cavity by endoscopic DCR. In individuals at high risk for nasolacrimal system involvement like patients whose conjunctival mass has spread to the medial canthus or involved the punctums and caruncles, or patients with symptomatic nasolacrimal duct obstruction following conjunctival mass removal, endoscopic nasal and lacrimal exams in regular follow-ups are advisable. Due to the unclear mechanism of delayed lacrimal system melanoma after the CM treatment, it is crucial to be diagnosed as soon as possible. Recent case series showed that patients with nonbulbar conjunctival melanoma should visit an ophthalmologist immediately in case of nose bleeding and recurrent sign of nose obstruction, because patients often did not report these symptoms which may cause delay in treatment [19]. The significance of symptoms like epiphora, epistaxis, and nasal blockage as the common symptoms of delayed nasolacrimal system melanoma is usually ignored by the patients. So, it is necessary to inform and instruct them about nasal symptoms and have them return to their ophthalmologist. Likewise, ophthalmologists must be vigilant of possible nasolacrimal involvement even if there is no obvious ocular recurrence for years after the CM treatment. Long-term follow-up is essential as a previous case report showed the recurrence after 21 years [20]. Furthermore, as molecular and genetic knowledge advances, there may be improvements in targeted therapeutic options and methods for preventing disseminated disease.

## Figures and Tables

**Table 1:** Reported cases of conjunctival melanoma with metastasis to lacrimal drainage system.

Author/year/type of article	Age & sex	Type of intervention	Site of metastasis
Rao et al. [21] Case report	42 Y/O Male	Excise dacryocystectomy	Nasolacrimal duct

Satchi et al. [12] Case series	81 Y/O Female	Orbital exenteration	(i) Upper lacrimal cannaliculus (ii) Lacrimal sac
	57 Y/O Male		(i) Lacrimal sac (ii) Upper nasolacrimal duct
	60 Y/O Male		Lacrimal sac
	25 Y/O Female		(i) Lacrimal sac (ii) Upper nasolacrimal duct
	58 Y/O Female		Lacrimal sac

McNab and Mackelvie [22] Case report	81 Y/O Female	Exenteration	Lacrimal sac

## Additional Points

*Précis*. This case represents a nasal cavity and lacrimal drainage system melanoma without punctual involvement following complete excision of ipsilateral conjunctival melanoma.
